# Hormonal therapy or chemotherapy for advanced endometrial cancer? A mixed-methods study assessing physicians’ attitudes

**DOI:** 10.1007/s00404-026-08486-0

**Published:** 2026-06-12

**Authors:** Willem Jan van Weelden, Julia Mesu, Petronella B. Ottevanger, Johanna M.A. Pijnenborg, Johanna W.M. Aarts

**Affiliations:** 1 Department of Obstetrics and Gynaecology, Canisius Wilhelmina Hospital, Nijmegen, The Netherlands; 2 Department of Obstetrics and Gynecology, Radboud university medical center, Geert Grooteplein Zuid 10, 6525 GA Nijmegen, The Netherlands; 3 Faculty of Medicine, Maastricht University, Maastricht, The Netherlands; 4 Department of Medical Oncology, Radboud university medical center, Nijmegen, The Netherlands; 5 Department of Obstetrics and Gynaecology, Cancer Center Amsterdam, Amsterdam University Medical Centers, Amsterdam, The Netherlands

**Keywords:** Endometrial cancer, Palliative treatment, Hormonal therapy, Chemotherapy, Physician’s attitudes

## Abstract

**Purpose:**

Hormonal therapy and chemotherapy are common systemic therapy options in advanced and recurrent endometrial cancer. There is limited consensus on factors that affect therapy selection. This study aims to gain insight into those factors at the level of clinical decision-making and at the level of patient- and tumor-specific factors.

**Methods:**

We performed an international cross-sectional study among medical oncologists and gynecologists. The survey consisted of (1) statements about barriers and facilitators that affect the process of decision-making and (2) hypothetical vignette cases investigating three patients and tumor factors that were tested with three levels each. Results were analyzed with descriptive statistics and linear mixed-effects models.

**Results:**

167 physicians responded to the survey. The most important facilitating factors were: understanding the patient’s personal context and the role of multidisciplinary tumor board discussions. The most important barrier was the lack of evidence for prediction of response to hormonal therapy and chemotherapy. The three patient and tumor factors included in the vignette cases were severity of symptoms, performance status, and progesterone-receptor (PR) expression in the tumor. PR expression and performance status were most discriminative, with high PR expression and poor performance status favoring hormonal therapy.

**Conclusion:**

The provision of personalized care in palliative treatment of endometrial cancer can be improved by multidisciplinary tumor board discussions with the personal context and preference of the patient in mind. The patient factors, severity of symptoms and WHO performance status, and the tumor factor, PR tumor expression, were most relevant for selecting hormonal therapy or chemotherapy.

**Supplementary Information:**

The online version contains supplementary material available at 10.1007/s00404-026-08486-0.

## What does this study adds to the clinical work

**Table:** 

The selection of palliative systemic therapy in endometrial cancer depends on progesterone receptor expression in tumor cells and performance status of the patient. Close collaboration between professionals in multidisciplinary tumor boards was the most important facilitator for provision of good clinical care.	

## Introduction

Therapy selection in patients with cancer is increasingly complex as more and more treatment options become available. Especially, in palliative medicine, it is essential to balance treatment benefits and side-effects and quality of life. Although endometrial cancer (EC) is curable in most patients, about 100,000 women are annually diagnosed with incurable EC worldwide [1]. For these, women palliative chemotherapy and hormonal therapy are the most common systemic treatment modalities [2, 3]. Chemotherapy yields a tumor response in around 50% of cases, but is associated with significant toxicity, causing 30% of patients to stop treatment [4, 5]. Hormonal therapy has response rates of 15–33% in unselected populations, but severe side-effects are uncommon, occurring in 6% of treated women [6, 7]. In the last decade, several new tumor-targeted treatment options in EC have been introduced which have high potential in selected patients, yet these therapies have substantial side-effects in more fragile patients [8, 9]. This results in application of novel immunotherapy drugs in just 15% of EC patients [10]. Currently, it is not clear which treatment should be preferred nor is it completely clear which factors predict response. Tumor factors that currently play a role are mismatch repair (MMR) status, hormone receptor status, and tumor grade. Hormone receptor-positive, low-grade tumors are more sensitive to hormonal therapy and MMR deficiency predicts response to immunotherapy [2, 11]. So far, it is unclear which patient factors impact the physicians’ decision in selecting a systemic therapy. This might lead to treatment choices that are not tailored to the patient’s specific situation resulting in suboptimal, non-patient-centered care. Therefore, we investigated factors that physicians use to select the correct treatment on the level of physicians’ decision-making, including barriers and facilitators that affect the process of therapy selection, and on the level of clinical patient and tumor factors that are used for palliative systemic treatment selection. This is relevant for hormonal therapy and chemotherapy, but also provides insight into patient factors that are relevant for novel drugs in the palliative setting.

## Materials and methods

We performed a survey among an international group of gynecologists and medical oncologists using a mixed-methods study design. This study was performed in accordance with the Declaration of Helsinki and approved by the institutional review board of the organizing medical center (approval number 2017-3803).

### Participants

Participants were physicians treating patients with advanced or recurrent EC. The survey was disseminated among various professional gynecological cancer networks including the EORTC-GCG (European Organisation for Research and Treatment of Cancer–Gynecological Cancer Group), DGOG (Dutch Gynecological Oncology Group), NSGO (Nordic Society of Gynecological Oncology), WOG (Dutch Working Group Gynecologic Oncology), ENITEC (European Network of Individual Treatment in Endometrial Cancer), ASGO (Australian Society of Gynecologic Oncologists), ANZGOG (Australia New Zealand Gynecological Oncology Group), NRG Oncology, and ESGO (European Society of Gynecological Oncology). The survey was disseminated between April 12th 2021 and April 30th 2021.

### Survey development

**Fig. 1:**
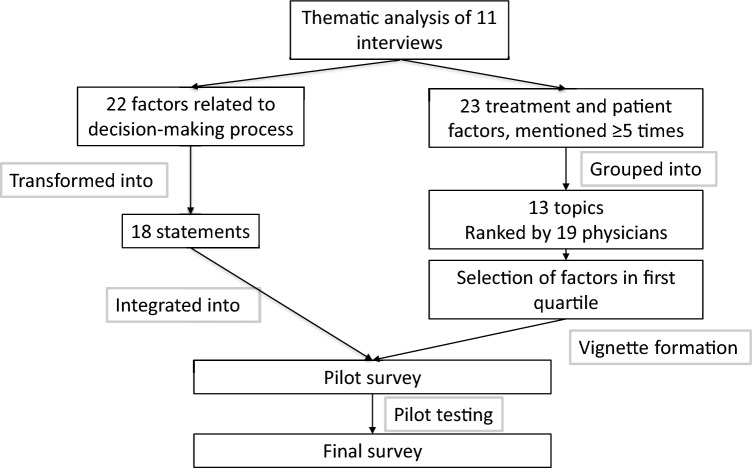
On the left, the process of developing the statements is shown. On the right, the development of the vignette study is shown

The survey consisted of two parts: (1) statements to identify barriers and facilitators in the process of clinical decision-making regarding treatment choice and (2) a vignette study to identify the most relevant patient and tumor factors that affect therapy selection. An overview of the development of the survey is shown in Fig. 1.

First, we performed 11 interviews with medical oncologists (*n* = 7) and gynecologists (*n* = 4) to generate input for the survey from members of the ENITEC network. Participants in the interviews were purposively sampled to include physicians working in general hospitals and university hospitals in different European countries with a range in years of experience. The interviews took place around the introduction of immunotherapy in clinical practice for EC. As a consequence, immunotherapy was not incorporated as palliative treatment for the vignette study. Interviews were transcribed verbatim and a thematic analysis was performed independently by two researchers (WW, JM). Intercoder reliability was established by repeated comparison and restructuring of codes used. From the interviews, we identified 22 factors related to the process of decision-making. The factors were subsequently transformed into 18 statements (see Online Resource 1). For the vignette study on patient and tumor factors, we first identified from the interviews 23 factors that were mentioned by five or more physicians (see Online Resource 2). The 23 factors consisted of 13 different subjects. The subjects were subsequently renamed and then ranked for importance by 8 medical oncologists and 11 gynecologists. The mean ranking score for each of the 13 factors was calculated and a quartile calculation was applied (Online Resource 3). As the number of vignettes increases drastically with each extra factor included, we decided to include the factors with a mean score within the first quartile: severity of symptoms of a patient, WHO performance status and progesterone-receptor (PR) status of the tumor***.*** We developed the vignettes as representative clinical scenarios that may occur in daily practice [12]. To that end, the three factors were each assigned three levels which represented a degree to which a factor may be present or absent in clinical practice (see Online Resource 4 for factors and levels).

The combination of three behavior factors, each consisting of three levels, resulted in 27 (3^3^) possible scenarios. We used an orthogonal main effects design following the methodology of Koedoot et al. [12]. This provides a subset of all possible combinations of characteristics and allows estimations of the relative weights for each level of the presented characteristics on the preference score. For each vignette, respondents rated their strength of preference for prescribing hormonal therapy on an anchored 7-point scale ranging from a strong preference for chemotherapy (score = 1) via a neutral position (score = 4) to a strong preference in favor of hormonal therapy (score = 7).

The final survey, including statements to identify barriers and facilitators and vignette cases, was pilot-tested among direct colleagues, resulting in some minor adjustments. The complete survey can be found in Online Resource 5.

### Statistical analysis

Statistical analysis was performed using different methods for the statements and vignettes. Descriptive statistics were used for the analysis of the statements. Frequencies of the answers “agree”, “neutral”, and “disagree” were counted for each statement for all participants, as well as separately for gynecologists and medical oncologists to study interprofessional differences.

The strength of preference for each vignette was analyzed using a mixed linear model with the preference score as a dependent variable and the attributes as factors. The model used a random intercept per participant. With estimates of fixed effects, preferences to prescribe chemotherapy or hormonal therapy were calculated relative to a reference level of the variable. Absolute preferences to any systemic therapy were calculated with estimated marginal means. The analyses were performed in SPSS version 28 using the linear mixed-effects model function (procedure MIXED). Statistical uncertainty was expressed in 95% confidence intervals (CIs). For a detailed explanation of the chosen statistical analysis of the vignette study, see Online Resource 6**.**

## Results

### Participants

A total of 167 physicians responded to the survey, including 100 (59.9%) gynecologists and 67 (40.1%) medical oncologists. The response rate could not be determined as the survey was spread through different and partially overlapping professional networks. Respondents were mostly from the Netherlands (35.9%) or other European countries (55.1%). Nine percent of participants practiced outside of Europe (Australia, USA, and Surinam). The characteristics of the participants can be found in Table 1.

**Table 1:** information of participant to survey

Characteristics	*n*=167(%)
Total participants	
Medical oncologists	67 (40.1%)
Gynecologists	100 (59.1%)
Country of practice	
The Netherlands	60 (35.9%)
European countries	92 (55.1%)
Other	15 (9.0%)
Type of practice	
University hospital	98 (58.7%)
General hospital	50 (29.4%)
Private practice	6 (3.6%)
Other (oncology center, multiple etc.)	13 (7.8%)
Network memberships*	
ENITEC	17 (10.2%)
EORTC	64 (38.3%)
DGOG	28 (16.8%)
WOG	52 (31.1%)
NSGO	29 (17.4%)
ASGO	5 (3.0%)
ANZGOG	3 (1.8%)
NRG	11 (6.6%)
NCRI	13 (7.8%)
Other (ESGO, ESTRO BGOG, etc.)	36 (21.6%)

*Network memberships do not add up to 167 as individual participants often had multiple memberships. The percentage of participants reflects the proportion of respondents that are member of a network

### Statements to identify barriers and facilitators in process of clinical decision-making

**Fig. 2:**
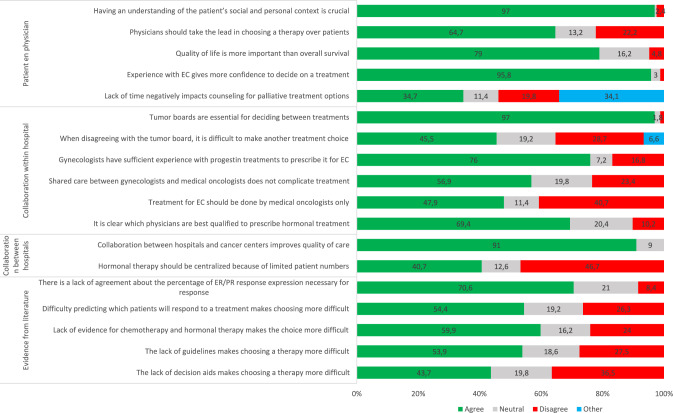
The answers that are shown are agree (green), neutral (gray), and disagree (red). For questions 5 and 7, there was an alternative option other (marked in blue). The answer option 'other' for statement 5 was 'I do not experience lack of time.' For statement 7, the other option was: 'physicians should not deviate from the advice of the tumor board'

The statement was analyzed on four levels: patient and physician, collaboration in hospital, collaboration between hospitals, and evidence from literature (see Fig. 2).

#### Patient and physician

For selecting a palliative systemic treatment option, almost all physicians agree that an understanding of the personal context of the patient is essential (97% agree). With that in mind, most physicians feel that they should take the lead in discussing treatment options (64.7% agree). Most respondents also agree that quality of life is more important than the effect of the therapy on survival (79% agree). From a physician’s perspective, experience with EC treatment is very important in selecting a treatment (95.8% agree). Lack of time for discussing options negatively affects the treatment decision for 34.7% of respondents.

#### Collaboration within the hospital

The results endorse the importance of multidisciplinary tumor board meetings (97% agree). A disadvantage of a tumor board might be that physicians find it difficult to deviate from the tumor board advice. 45.5% of physicians recognize this difficulty. As for who should prescribe hormonal therapy: 76% of respondents agree that gynecologists have sufficient experience to prescribe progestin therapy, the most common form of hormonal therapy. Among gynecologists, 84% agreed with this statement; among medical oncologists, this was 64.2%. On the other hand, 47.9% of respondents agree that systemic therapy should be prescribed only by medical oncologists. Among medical oncologists, 64.2% agreed with this statement; for gynecologists, this was 37%. Nevertheless, 56.9% of respondents indicate that shared care between gynecologists and medical oncologists does not complicate treatment: this was 63% for gynecologists and 47.4% for medical oncologists.

#### Collaboration between hospitals

According to 91% of respondents, collaboration between hospitals is essential to select the optimal palliative systemic therapy. Whether hormonal therapy should be centralized because of small numbers is less unanimous: 40.7% agree with this statement.

#### Evidence from literature

70.6% of respondents indicate that there is a lack of agreement about the percentage of estrogen and progesterone-receptor expression necessary for response. A majority of 59.9% also agree that a lack of evidence for chemotherapy and hormonal therapy causes therapy selection to be more difficult. Whether a lack of guidelines or a decision aid makes therapy selection more difficult is not uniformly clear with 53.9% of respondents that would be helped with a guideline and 43.7% of respondents that would need a decision aid.

### Vignette study

The preference in prescribing chemotherapy or hormonal therapy was explored with a vignette study. The three included factors were severity of symptoms, progesterone-receptor expression, and performance status. These three factors were tested at three different levels each (see Online Resource 4 for overview levels of factors). All three factors significantly impacted therapy selection (*p* < 0.001). The preference for hormonal therapy or chemotherapy was scored on a scale from −3 to + 3: a score of 0 indicates no preference, a score > 0 indicates a preference for chemotherapy, and a score < 0 indicates a preference for hormonal therapy.

**Fig. 3:**
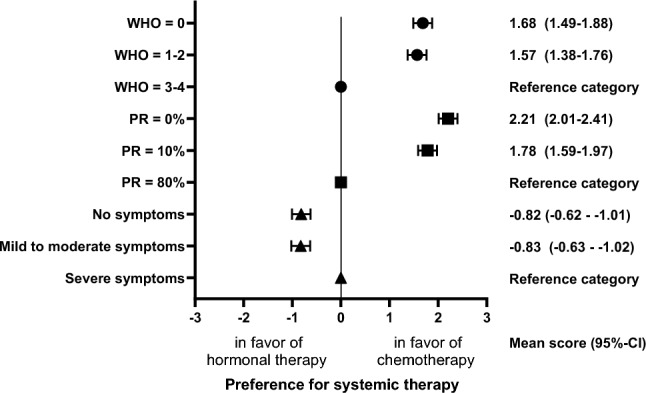
The relative scores of the level of each variable are shown compared to a reference level of the same variable. Scores are shown as average scores (indicated by a black circle, square, or triangle) with 95% confidence interval estimates (shown by a bar)

In Fig. 3, the preference for prescribing hormonal therapy or chemotherapy is shown for the level of each variable compared to a reference level of the same variable. For WHO performance status, the reference level is WHO 3–4, indicating a condition in which limited or no self-care can be done. Compared to WHO 3–4, physicians have a preference to prescribe chemotherapy in patients with a good performance status (WHO = 0) as indicated by the score of + 1.68 (95%CI 1.49–1.88). In patients with a WHO performance status of 1–2, physicians still prefer chemotherapy (mean score of 1.57 (95%CI 1.37–1.76)).

The scores do not indicate an absolute preference for prescribing a therapy. If an average for all other factors is assumed, the mean score for physicians can be calculated on a 1–7 scale with 3.5 as neutral score, a score > 3.5 as preference for chemotherapy, and a score < 3.5 as preference for hormonal therapy. Physicians have a preference for chemotherapy in case of WHO 0 (mean score 4.32, (95%CI 4.15–4.49) and WHO 1–2 (mean score 4.20, (95%CI 4.03–4.38)), and a clear preference to prescribe hormonal therapy in patients with WHO 3–4 performance status (mean score 2.64, (95%CI 2.46–2.81).

**Fig. 4:**
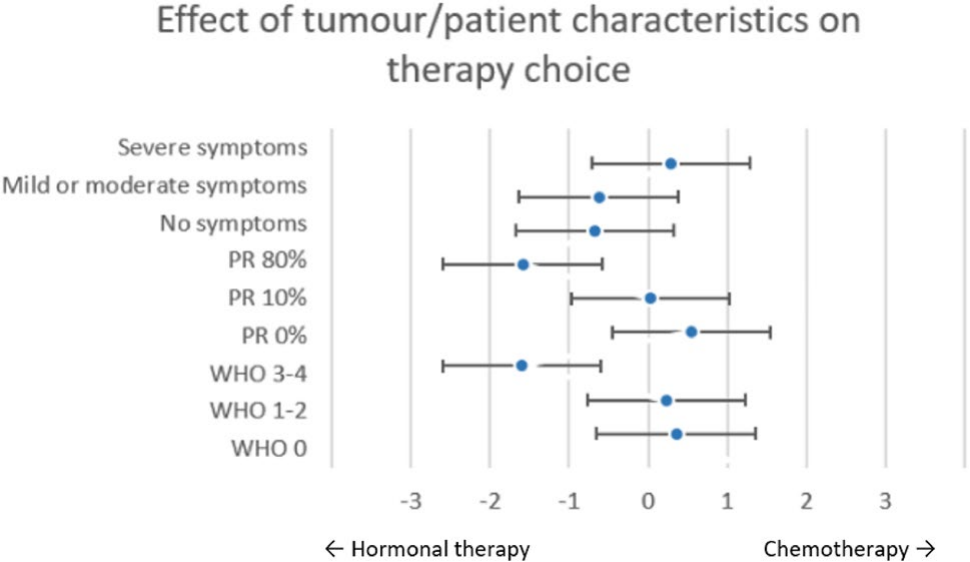
How different levels of patient and tumor factors impact prescription behavior. The error bars display the 95% confidence interval

Figure 3 also demonstrates preference to prescribe chemotherapy or hormonal therapy in relation to PR expression, with PR 80% as reference level. Compared to the reference level PR80%, there is a preference chemotherapy in case PR expression is 0% or 10%. Also in absolute terms, there is a preference to prescribe chemotherapy in case of PR 0% (mean score 4.60 (95%-CI 4.42–4.77) and PR 10% (4.17 (95%-CI 4.00–4.35 on a scale from 1 to 7). In case of PR, 80% physicians report a clear preference to prescribe hormonal therapy [score 2.39 (95%-CI 2.22–2.56) on a scale from 1 to 7] (Fig. 4).

For severity of symptoms, the reference level is severe symptoms. Compared to severe symptoms, physicians have a preference to prescribe hormonal therapy in case of no symptoms or mild or moderate symptoms. In absolute terms, physicians have no clear preference to prescribe chemotherapy or hormonal therapy in case of no or mild-to-moderate symptoms (no symptoms: score 3.45 (95%CI 3.28–3.62), mild-to-moderate symptoms: score 3.44 (95%CI 3.27–3.61) on a scale from 1 to 7).

## Discussion

In this international cross-sectional study, we evaluated the decision-making process for palliative systemic therapy in endometrial cancer on the level of barriers and facilitators for therapy selection and at the level of patient and tumor factors.

One important facilitator is knowing the social and personal context of the patient. With this information in mind, most physicians feel that they should take the lead in the clinical consultation on systemic therapy selection. Exploring the patient’s preferences and values is needed prior to integration into the final treatment decision [13].

Multidisciplinary tumor board meetings are other facilitators for almost all respondents, and allow consultations and discussions with experts from different medical specialties [14]. For endometrial cancer, this includes gynecologists, medical oncologists, radiation oncologists, pathologists, and radiologists. Multidisciplinary consultations are recommended in patients with stage III/IV endometrial cancer and are considered a quality indicator for provision of optimal care in endometrial cancer [2]. From a patients’ perspective, multidisciplinary tumor board meetings result in more accurate surgical and systemic treatment recommendations [15].

Most respondents agree that gynecologists can prescribe progestin type hormonal therapy.

Yet, the majority of medical oncologists believe that they should prescribe all palliative systemic therapies. This might be a reflection of the local situation in some countries where medical oncologists prescribe all types of palliative systemic therapy with the exception of progestin hormonal therapy. Prescription of chemotherapy and hormonal therapy differs within countries and includes gynecologists and medical oncologists prescribing both therapies and medical oncologists prescribing all types of palliative systemic treatments. Although gynecologists are familiar with hormonal therapy, most do not prescribe chemotherapy and immunotherapy. The increasing number of targeted treatment options questions if systemic treatment consultations should not be performed by the medical oncologist alone.

We hypothesized that hormonal therapy administration might be best centralized in a limited number of hospitals, but this was not confirmed in the survey: centralization is not necessary according to most respondents. Tumor board discussions between hospitals might be an alternative for centralization.

Finally, there was a majority of respondents who agreed to a lack of evidence to predict response to chemotherapy and hormonal therapy. However, this did not unequivocally lead to difficulty in prescribing treatment. Most respondents were not hindered in therapy selection by the lack of evidence to predict response to therapy.

Interestingly, from the vignette study, we learned that severity of symptoms did not affect therapy selection as much as PR expression and WHO performance status. Severity of symptoms affected preference for hormonal therapy to some extent in the case of limited or no symptoms. WHO performance status resulted in clear changes in preference for hormonal therapy or chemotherapy. WHO performance status 3–4 indicates a condition in which the patient can only do limited or no self-care and is confined to a chair or bed for more than 50% of the time. The presence of WHO performance status 3–4 resulted in a preference for hormonal therapy, likely because chemotherapy is not a realistic treatment option in these patients. Application of chemotherapy for patients at the end of life with distant metastases or disease refractory to at least 1 line of chemotherapy does not provide any improvement in quality of life and should, therefore, not be used routinely [16].

Physicians reported that the most important reasons for preferring hormonal therapy include: fewer side-effects and fewer hospital visits of hormonal therapy and superior effectivity of hormonal therapy as first-line therapy (as opposed to second or third line therapy). In the case of PR expression of 80% in tumor tissue, there was a clear shift in preference toward hormonal therapy. PR expression is a predictive marker for response and response duration to hormonal therapy with higher efficacy in cases with positive PR expression [17, 18]. However, a cut-off point to differentiate between positive and negative PR expression is unclear. In a recent study, response increased proportionally from 0% in PR expression 0–50% to 67% in the case of PR expression >70% [6]. However, in earlier studies, response to hormonal therapy in the case of negative or low PR expression was reported [17]. This might explain why PR 0% and PR 10% cut-offs yield similar preferences toward chemotherapy as observed in this study. Since execution of this study, other predictive (molecular) markers have been identified to correlate with response to specific therapies. In particular, MMR status clearly correlates with response to immunotherapy, making immunotherapy first-line treatment as palliative systemic therapy in MMR-deficient tumors. In the rapidly evolving landscape of systemic therapy in EC, other therapies like PARP inhibitors in tp53-mutated tumors and trastuzumab for tumors with overexpression of HER2 can now be considered in selected groups as palliative systemic therapy [19]. With the introduction of these therapies, decision-making in selecting systemic palliative therapy will become more complex. Results of this study show that patient factors like WHO performance status and severity of symptoms should not be overlooked in the process of treatment selection.

This is the first study that assessed the clinical decision-making process of physicians for the choice of systemic palliative treatment in EC patients. Strengths of this study include the step-wise approach starting with interviews to elicit the most important themes to development of the statements and vignettes. There are also some limitations that need to be addressed. Most respondents were European, which could limit external validity outside Europe. Furthermore, a vignette study is a good way to mimic real prescription behavior, but it remains a simplified representation with a limited number of factors to keep the survey short enough to obtain enough responses. Other factors like patient preference, expected time to clinical response, and side-effects were found to be relevant, but could not be included in the vignette.

## Conclusion

On the level of physicians’ decision-making for therapy selection in palliative systemic treatment of endometrial cancer, the most important factors that facilitated provision of clinical care were: knowing the context of the patient and multidisciplinary tumor board meetings. With close collaboration between gynecologists and medical oncologists and between different hospitals, a majority of respondents agreed to share care of these patients between gynecology and oncology with no further need for centralization to a small number of hospitals. Severity of symptoms and WHO performance status were the most important patient factors that affected therapy selection. PR expression was the most important tumor factor for therapy selection at the time of execution of this study. These results provide useful insights into the decision-making process for treatment allocation from the perspective of the physician and provide relevant patient factors for selection of systemic palliative therapy in EC.

## Supplementary Information

Below is the link to the electronic supplementary material.

Supplementary file1 (DOCX 16 KB)

Supplementary file2 (DOCX 15 KB)

Supplementary file3 (DOCX 49 KB)

Supplementary file4 (DOCX 12 KB)

Supplementary file5 (DOCX 41 KB)

Supplementary file6 (DOCX 24 KB)

## Data Availability

All data generated and analyzed during this study are included in this article.
