# Supplementary material for: Hormonal therapy or chemotherapy for advanced endometrial cancer? A mixed-methods study assessing physicians’ attitudes
Source: Arch Gynecol Obstet. 2026 Jun 12;313(1):254. doi: 10.1007/s00404-026-08486-0 (PMC13481385; doi:10.1007/s00404-026-08486-0)
Supplement: Supplementary file 1 [file 404_2026_8486_MOESM1_ESM.docx]

**Online Resource 1**

|  | ***Factors related to decision making*** | ***Statements*** |
| --- | --- | --- |
| *1* | There are different opinions about the degree of ER/PR expression needed for response to hormonal therapy. | To me the patient’s quality of life is more important than overall survival. |
| *2* | It is difficult to predict which patients are going to experience a response to hormonal therapy. | Tumour boards (multidisciplinary meeting with different specialities) are an essential part of deciding on the best treatment choices for my patient. |
| *3* | There is a lack of evidence. | Whenever I disagree with the treatment recommendation given by the tumour board, I don’t find it difficult to make a different choice for my patient. (If you are of the opinion that physicians should not deviate from this advice, please opt disagree and state this in the comment section below.) |
| *4* | Sometimes, it is difficult to determine the order in which to try different therapies. | It is crucial for me to have an understanding of the personal and social context of the patient in order to have a proper discussion about treatment options. |
| *5* | There is a lack of practice recommendations/commonly occurring scenarios. | The more experienced I become in treating patients with endometrial carcinoma, the more confident I become at choosing the best treatment with my patient. |
| *6* | Quality of life is just as important as survival. | 6. I think gynaecologists have sufficient experience with prescribing progestin treatment to patients with recurrent or advanced endometrial carcinoma. |
| *7* | Patients leave therapy decision up to physician. | 7. In my practice, lack of time negatively impacts palliative counselling for the treatment options chemotherapy or hormonal therapy. (Please specify in comments if this is currently not the case in your place of practice). |
| *8* | Good communication between gynaecologists and medical oncologists facilitates good palliative therapy. | . I think close collaboration between general hospitals and specialised oncology centres improves the quality of care for patients. |
| *9* | Space to follow or discard tumour board advice is important. | There is a lack of agreement about the percentage of ER/PR receptor positivity that is needed for hormonal therapy to be effective. |
| *10* | Tumour board meetings are an important part of making a treatment decision. | I find it difficult to predict which patients will respond to hormonal therapy which limits my ability to make suitable treatment choices. |
| *11* | It is unclear what medical oncologists prescribe in general hospitals. | There is a lack of evidence for treatments hormonal therapy and chemotherapy for advanced and recurrent endometrial carcinoma which limits my ability to make suitable treatment choices. |
| *12* | Being familiar with the patient and her values facilitates making a suitable treatment choice. | I think physicians should take the lead in deciding the proper therapy for individual patients, because patients often don’t know about available treatment options. |
| *13* | Being experienced in treating advanced or recurrent EC facilitates making a suitable treatment choice. | Sharing the care of patients who receive hormonal therapy by medical oncologists and gynaecologists complicates the treatment of these patients unnecessarily. (If in your centre hormonal therapy is solely prescribed by gynaecologist or medical oncologist, please select disagree and state which specialist prescribes hormonal therapy in your centre). |
| *14* | Gynaecologists are sufficiently experienced in treating patients with hormonal therapy. | I find it clear which physicians are best qualified to safely prescribe and monitor hormonal therapy. |
| *15* | Preference of physician is deciding in treatment choice. | I think that due to the limited number of patients that might be eligible for hormonal therapy, this care should be centralised to improve the quality of patient care. |
| *16* | Medical oncologists are more experienced in treating patients with hormonal therapy. | I think systemic treatment for advanced or metastatic endometrial carcinoma should be exclusively provided by medical oncologists because they are more experienced than gynaecologists. |
| *17* | Counselling should be aimed at palliative care and consultations should be sufficiently long to do so | The lack of properly defined guidelines makes it more difficult to choose a suitable treatment for my patient |
| *18* | Physician prescribing therapy should also be responsible for follow-up care | The lack of decision aids makes it difficult to select a suitable treatment option with my patient. |
| *19* | There should be good communication between general and university hospitals |  |
| *20* | Gynaecology and medical oncology share patients receiving hormonal therapy |  |
| *21* | It is not clear which physician is certified to prescribe certain treatments |  |
| *22* | There are few patients per centre |  |
