# Supplementary material for: Hormonal therapy or chemotherapy for advanced endometrial cancer? A mixed-methods study assessing physicians’ attitudes
Source: Arch Gynecol Obstet. 2026 Jun 12;313(1):254. doi: 10.1007/s00404-026-08486-0 (PMC13481385; doi:10.1007/s00404-026-08486-0)
Supplement: Supplementary file 2 [file 404_2026_8486_MOESM2_ESM.docx]

**Online Resource 2**

|  | ***Patient and tumour factors mentioned ≥5 times*** | ***Grouping of patient and tumour factors*** |
| --- | --- | --- |
| *1* | Importance ER/PR expression: unclear which therapy to prescribe in case of low PR and/or high ER expression | Severity of symptoms |
| *2* | Unknown which degree of ER/PR receptor expression corresponds with response to hormonal therapy | Hormone receptor status of tumour |
| *3* | Unclear whether to prescribe thrombotic prophylaxis when prescribing progestins | WHO performance status |
| *4* | There are no best practices/commonly occurring scenario’s | Patients’ preference |
| *5* | The guidelines are unclear | Expected time until clinical response |
| *6* | It is difficult to determine the order of therapy | Side-effects of therapy |
| *7* | With hormonal therapy, it takes longer before clinical effect is seen than with chemotherapy | Tumour grade (degree of differentiation) |
| *8* | It is unclear which patients will and will not respond to hormonal therapy | Supporting evidence for treatment |
| *9* | If there is response to hormonal therapy, it is often long lasting | Age of patient |
| *10* | If there’s no response to hormonal therapy, chemotherapy can be attempted | Location of metastases (e.g. lungs or abdomen) |
| *11* | Comorbidities of patient are important in the choice of therapy | Size of tumour |
| *12* | General condition of the patient | Risk of venous thrombosis with hormonal therapy |
| *13* | Patient with severe symptoms 🡪 chemotherapy | Number of hospital visits required for treatment |
| *14* | Patient preference is important in therapy choice |  |
| *15* | In case of cardiovascular comorbidities, hormonal therapy might be more risky |  |
| *16* | Age of the patient plays a role in therapy choice |  |
| *17* | In lung metastases, patient symptoms determine choice of therapy |  |
| *18* | Tumours of grade 1 and 2 have a higher chance of responding to hormonal therapy |  |
| *19* | Size of tumour important when vital structures are at risk |  |
| *20* | Lung metastases might respond to hormonal therapy just as well as abdominal metastases |  |
| *21* | Side effects of hormonal therapy are more bearable than those of chemotherapy |  |
| *22* | There needs to be a balance between effects and side effects of therapy |  |
| *23* | Hormonal therapy is less burdensome than chemotherapy (tablets at home vs. outpatient treatment at hospital) |  |
