# Supplementary material for: Hormonal therapy or chemotherapy for advanced endometrial cancer? A mixed-methods study assessing physicians’ attitudes
Source: Arch Gynecol Obstet. 2026 Jun 12;313(1):254. doi: 10.1007/s00404-026-08486-0 (PMC13481385; doi:10.1007/s00404-026-08486-0)
Supplement: Supplementary file 3 [file 404_2026_8486_MOESM3_ESM.docx]

Online Resource 3

Mean ranking score of all factors

|  |  |
| --- | --- |
| Number of hospital visits required for treatment | 11.26 |
| Risk of venous thrombosis with hormonal therapy | 10.79 |
| Size of tumour | 10 |
| Location of metastases (e.g. lungs or abdomen) | 8.32 |
| Age of patient | 7.84 |
| Supporting evidence for treatment | 7.47 |
| Tumour grade (degree of differentiation) | 7.05 |
| Side effects of therapy | 6.89 |
| Expected time until clinical response | 5.58 |
| Patients' preference | 4.26 |
| WHO performance status | 4.16 |
| Hormone receptor status of tumour | 3.89 |
| Severity of symptoms of patient | 3.47 |

|  | Q1 = 4.21 |
| --- | --- |
| 4.21 | 0 |
| 4.21 | 1 |
