# Supplementary material for: Hormonal therapy or chemotherapy for advanced endometrial cancer? A mixed-methods study assessing physicians’ attitudes
Source: Arch Gynecol Obstet. 2026 Jun 12;313(1):254. doi: 10.1007/s00404-026-08486-0 (PMC13481385; doi:10.1007/s00404-026-08486-0)
Supplement: Supplementary file 4 [file 404_2026_8486_MOESM4_ESM.docx]

Online Resource 4

|  | Progesterone receptor expression | WHO performance status | Severity of symptoms |
| --- | --- | --- | --- |
| Level 1 | 80% expression | 0 | No symptoms |
| Level 2 | 10% expression | 1-2 | Moderate symptoms with low impact on quality of life |
| Level 3 | 0% expression | 3-4 | Severe symptoms with great impact on quality of life. |
