# Supplementary material for: Hormonal therapy or chemotherapy for advanced endometrial cancer? A mixed-methods study assessing physicians’ attitudes
Source: Arch Gynecol Obstet. 2026 Jun 12;313(1):254. doi: 10.1007/s00404-026-08486-0 (PMC13481385; doi:10.1007/s00404-026-08486-0)
Supplement: Supplementary file 5 [file 404_2026_8486_MOESM5_ESM.docx]

Online Resource 5

## Start of survey

Prior to the start of this survey, please answer the following questions.

A. I currently practice as a

- Gynaecologist or Gynaecological oncologist
- Medical oncologist
- Other [	]

B. Country of current practice (drop down menu)
C. Type of centre in which practice takes place

- General hospital
- University hospital
- Private practice
- Other [	]

**Part A Attitude factors**

The following section consists of statements. For each of these, please select disagree, partly disagree, neither disagree nor agree, partly agree or agree.

1. To me the patient’s quality of life is more important than overall survival.

2. Tumour boards (multidisciplinary meeting with different specialities) are an essential part of deciding on the best treatment choices for my patient.

3. Whenever I disagree with the treatment recommendation given by the tumour board, I don’t find it difficult to make a different choice for my patient. (If you are of the opinion that physicians should not deviate from this advice, please opt disagree and state this in the comment section below.)

4. It is crucial for me to have an understanding of the personal and social context of the patient in order to have a proper discussion about treatment options.

5. The more experienced I become in treating patients with endometrial carcinoma, the more confident I become at choosing the best treatment with my patient.

6. I think gynaecologists have sufficient experience with prescribing progestin treatment to patients with recurrent or advanced endometrial carcinoma.

7. In my practice, lack of time negatively impacts palliative counselling for the treatment options chemotherapy or hormonal therapy. (Please specify in comments if this is currently not the case in your place of practice)

8. I think close collaboration between general hospitals and specialised oncology centres improves the quality of care for patients.

9. There is a lack of agreement about the percentage of ER/PR receptor positivity that is needed for hormonal therapy to be effective.

10. I find it difficult to predict which patients will respond to hormonal therapy which limits my ability to make suitable treatment choices.

11. There is a lack of evidence for treatments hormonal therapy and chemotherapy for advanced and recurrent endometrial carcinoma which limits my ability to make suitable treatment choices.

12.I think physicians should take the lead in deciding the proper therapy for individual patients, because patients often don’t know about available treatment options.

13. Sharing the care of patients who receive hormonal therapy by medical oncologists and gynaecologists complicates the treatment of these patients unnecessarily. (If in your centre hormonal therapy is solely prescribed by gynaecologist or medical oncologist, please select disagree and state which specialist prescribes hormonal therapy in your centre).

14. I find it clear which physicians are best qualified to safely prescribe and monitor hormonal therapy.

15. I think that due to the limited number of patients that might be eligible for hormonal therapy, this care should be centralised to improve the quality of patient care.

16. I think systemic treatment for advanced or metastatic endometrial carcinoma should be exclusively provided by medical oncologists because they are more experienced than gynaecologists.

17. The lack of properly defined guidelines makes it more difficult to choose a suitable treatment for my patient.

18. The lack of decision aids makes it difficult to select a suitable treatment option with my patient.

**Part B Behaviour factors**

Vignettes, version 3

In this vignette study, nine clinical scenarios are presented to determine which treatment factors are most important in selecting palliative systemic therapy in endometrial cancer. Previous interviews have shown that the factors ‘progesterone expression in tumour tissue’, ‘severity of symptoms of a patient’ and ‘WHO performance status’ are the most relevant treatment factors that clinicians use to determine if a patient receives hormonal therapy or chemotherapy. In the following scenarios, these factors will vary in level. We would like you to indicate your preference for or against hormonal therapy or chemotherapy for each of these clinical cases. The levels used for all three factors are described below.

## Vignettes variables and levels

**WHO performance status**

| WHO performance status 0 | The patient is fully active, able to carry out all pre-disease activities without restrictions. |
| --- | --- |
| WHO performance status 1 – 2 | The patient is restricted in physically strenuous activities, able to perform self-care, may be able to perform light work and up and about at least 50% of the time. |
| WHO performance status 3 – 4 | The patient is confined to bed or chair more than 50% of the time, can only perform very limited self-care or none at all. |

**Severity of Symptoms**

This factor looks at the severity of symptoms from the patient’s perspective.

| None to mild symptoms | The patient experiences no symptoms or very mild symptoms which she herself reports do not impact her quality of life |
| --- | --- |
| Moderately severe symptoms | The patient experiences moderate symptoms which she herself reports as only very mildly impacting her quality of life if at all |
| Severe symptoms | The patient experiences severe symptoms which she herself reports as having a great impact on her quality of life |

**PR receptor expression by tumour**

- 0%
- 10%
- 80%

Vignette example:

The following case is an example in order to get used to the vignette format:

A 70-year old woman presents at the outpatient clinic with a systemic recurrence of endometrial carcinoma. There are no curative treatment options available.

The WHO performance status is 0, she has severe symptoms and the PR expression is 0%

In the above scenario I would choose….

1) Now imagine this scenario but instead with the following variables having changed:

| WHO performance status | 3 – 4 |
| --- | --- |
| Severity of symptoms | Severe symptoms with great impact on quality of life |
| Hormone receptor status | PR 0% |

*In the above scenario I would choose (assuming the patient wants some form of treatment)…*

2) Now imagine this scenario but instead with the following variables having changed:

| WHO performance status | 0 |
| --- | --- |
| Severity of symptoms | Moderate symptoms but with low impact on quality of life |
| Hormone receptor status | PR 80% |

*In the above scenario I would choose (assuming the patient wants some form of treatment)…*

3) Now imagine this scenario but instead with the following variables having changed:

| WHO performance status | 0 |
| --- | --- |
| Severity of symptoms | Patient doesn’t report any complaints related to her EC |
| Hormone receptor status | 0% |

*In the above scenario I would choose (assuming the patient wants some form of treatment)…*

4) Now imagine this scenario but instead with the following variables having changed:

| WHO performance status | 1 – 2 |
| --- | --- |
| Severity of symptoms | Patient doesn’t report any complaints related to her EC |
| Hormone receptor status | PR 10% |

*In the above scenario I would choose (assuming the patient wants some form of treatment)…*

5) Now imagine this scenario but instead with the following variables having changed:

| WHO performance status | 3-4 |
| --- | --- |
| Severity of symptoms | Patient doesn’t report any complaints related to her EC |
| Hormone receptor status | PR 80% |

*In the above scenario I would choose (assuming the patient wants some form of treatment)…*

6) Now imagine this scenario but instead with the following variables having changed:

| WHO performance status | 0 |
| --- | --- |
| Severity of symptoms | Severe symptoms with great impact on quality of life |
| Hormone receptor status | PR 10% |

*In the above scenario I would choose (assuming the patient wants some form of treatment)…*

7) Now imagine this scenario but instead with the following variables having changed:

| WHO performance status | 1 – 2 |
| --- | --- |
| Severity of symptoms | Severe symptoms with great impact on quality of life |
| Hormone receptor status | PR 80% |

*In the above scenario I would choose (assuming the patient wants some form of treatment)…*

8) Now imagine this scenario but instead with the following variables having changed:

| WHO performance status | 1 – 2 |
| --- | --- |
| Severity of symptoms | Moderate symptoms but with low impact on quality of life |
| Hormone receptor status | PR 0% |

*In the above scenario I would choose (assuming the patient wants some form of treatment)…*

9) Now imagine this scenario but instead with the following variables having changed:

| WHO performance status | 3 – 4 |
| --- | --- |
| Severity of symptoms | Moderate symptoms but low impact on quality of life |
| Hormone receptor status | PR 10% |

*In the above scenario I would choose (assuming the patient wants some form of treatment)…*
