# Supplementary material for: Hormonal therapy or chemotherapy for advanced endometrial cancer? A mixed-methods study assessing physicians’ attitudes
Source: Arch Gynecol Obstet. 2026 Jun 12;313(1):254. doi: 10.1007/s00404-026-08486-0 (PMC13481385; doi:10.1007/s00404-026-08486-0)
Supplement: Supplementary file 6 [file 404_2026_8486_MOESM6_ESM.docx]

Online resource ***	**Explanation of the chosen statistical analysis of the vignette study**

For this study we followed the methodology as in the study of Koedoot. The outcome measure is a 7‑point scale (scores 1–7), and the data are analysed using a linear mixed model with the vignette attributes as categorical explanatory variables and patient/respondent as the random factor (i.e. the “level” in the sense of mixed‑effects models, not the level of an attribute). For each respondent, multiple vignettes are available, each representing a particular combination of attribute levels, along with the respondent’s rating of each vignette on the 7‑point scale.

The outcome is not strictly a continuous variable, as would be ideal for a mixed model. The discrete scores are therefore treated as rounded estimates from an underlying continuous distribution. This approach is also used by Koedoot and is common practice, even with scales of fewer than 7 points (e.g. 4‑ or 5‑point scales).

The model included only a random intercept. Some respondents are expected to be more inclined towards prescribing chemotherapy than others and therefore, on average across all vignettes, to give higher scores (assuming that a score of 7 corresponds to a strong preference for chemotherapy). In the simulations, patient characteristics were not included. Such characteristics are likely to influence the scores, but incorporating them would substantially increase model complexity. This would go beyond the purpose of this study.

Power calculations require assumptions regarding effect sizes and their variability. In the present simulations, an effect size of 1 point was targeted, representing a clinically relevant but not excessively large difference. The variability of this effect was set to 1. For all other attributes, the effect was set to 0, implying that, on average, there is no preference for any specific level of those attributes. The baseline score for respondents (the intercept in the model) was set at 4, with a standard deviation of 2. Thus, each respondent’s baseline score was drawn from a distribution with mean 4 and standard deviation 2. Respondents with a strong preference for chemotherapy will tend to have high overall scores (6 or 7), whereas respondents with low scores will have a strong tendency to choose hormonal therapy. A residual variance also had to be specified. A given individual will not necessarily provide the same response to a given vignette on different occasions, and two otherwise very similar individuals may respond differently to the same vignette. This residual standard deviation was set at 2.

true values.

These simulations necessarily rely on assumptions, and the resulting conclusions should therefore be interpreted with appropriate caution. Nevertheless, this mixed‑model approach applied to vignette data is considered to be a powerful method. Although it cannot be guaranteed that the assumptions fully reflect reality, substantial variability and uncertainty have been embedded in the simulation framework.
